# Miniaturization of Human iPSC‐derived neuron long‐term culture into high‐throughput screening format for AD biology and therapeutics discovery

**DOI:** 10.1002/alz70859_103184

**Published:** 2025-12-25

**Authors:** Yuhong Du, Ying Zhou, Min Qui, Elizabeth L Zoeller, Ranjita Betarbet, Benjamin Siciliano, Allan I. Levey, Haian Fu, Zhexing Wen, The Emory‐SAGE‐SGC‐Jax TREAT‐AD Center

**Affiliations:** ^1^ Emory University, Atlanta, GA USA; ^2^ Emory University School of Medicine, Atlanta, GA USA

## Abstract

**Background:**

Many emerging molecular targets are implicated in AD, prompting the need for models to efficiently and rapidly evaluate their functional importance. Development of human induced pluripotent stem cell (hiPSC) models has accelerated our ability to study these processes across AD‐relevant cell types. Advantages of hiPSC cultures include their ability to regenerate, receive disease‐specific alterations in isogenic backgrounds, and withstand experimental variations in a controlled setting. Conversely, stringent culture conditions and prolonged differentiation limit the utility of hiPSC‐derived models, especially in high‐density plates. Leveraging our expertise at the Emory‐Sage‐SGC‐Jax TREAT‐AD Center in developing hiPSC‐derived neuron cultures and miniaturization technologies for high‐throughput screening (HTS), we optimized the hiPSC‐derived neuron long‐term culture into a 384‐well HTS format. A panel of functional HTS and image‐based high‐content screening (HCS) assays were developed to rapidly evaluate many experimental variables, including target modulations and small molecule treatment.

**Methods:**

The hiPSC‐derived neuron cells were seeded into the 384‐well PDL‐coated plate and maintained in neuron maturation culture medium for 4 weeks. The medium was changed twice weekly before compound treatment. The cells were proceeded to various HTS/HCS assays, including live‐cell based, such as mitochondrial function, cell viability, oxidative stress; and/or fixed‐cell based, such as immunofluorescence (IF) staining for neuron morphology and synaptic

**Results:**

The optimization of hiPSC‐derived neuron culture conditions resulted in healthy, long‐term differentiated neurons in a 384‐well HTS format. Copious data across multiple phenotypes were obtained through a panel of multiplexed functional assays for matured neurons. The optimization of HTS/HCS functional assays led to robust assay performance, which allows the sensitive detection of phenotypic changes with small molecules and biological perturbations of the neurons.

**Conclusions:**

We optimized the long‐term hiPSC‐derived neuron culture into a 384‐well HTS format for modeling AD and developed a panel of functional HTS/HCS assays for screening small molecules and biological perturbagens. The results from screening a set of well‐annotated small molecules with known biological targets and activities will enable us to rapidly advance knowledge of AD targets in hiPSC‐derived, AD‐relevant cell types. Our HTS neuron long‐term culture system will make expanded screening with large compound libraries feasible to accelerate AD drug discovery.

